# Is surgical debridement necessary in the diabetic foot treated with photodynamic therapy?

**DOI:** 10.1080/2000625X.2017.1373552

**Published:** 2017-09-19

**Authors:** João Paulo Tardivo, Rodrigo Serrano, Lívia Maria Zimmermann, Leandro Luongo Matos, Mauricio S. Baptista, Maria Aparecida Silva Pinhal, Álvaro N. Atallah

**Affiliations:** ^a^ Hospital Anchieta, Faculdade de Medicina ABC, São Bernardo do Campo, Brazil; ^b^ Departamento de Bioquímica, Faculdade de Medicina ABC, Santo André, Brazil; ^c^ Departamento de Bioquímica, Universidade de São Paulo, São Paulo, Brazil; ^d^ Departamento de Medicina, Universidade Federal de São Paulo, São Paulo, Brazil

**Keywords:** Diabetic foot, debridement, osteomyelitis, amputation, photodynamic therapy

## Abstract

**Background**: Diabetic patients are susceptible to developing foot ulcers with serious complications such as osteomyelitis and amputations. Treatment approaches are still empirical and the benefit of usual procedures such as surgical debridement has not been properly evaluated. Photodynamic Therapy (PDT) is a non-invasive and highly efficient method for the treatment of the diabetic foot, being able to eradicate the infection and to stimulate healing, decreasing considerably the amputation risk. In the day-to-day practice of our service, we have been faced with the question whether debridement is necessary before PDT. In here, we designed a study to answer that question.

**Methods**: Patients were divided in two groups: In one of the groups (n = 17), debridement was performed before PDT and in the other (n = 40) only PDT treatment was performed. PDT sessions were performed once a week in all patients until healing was achieved, as indicated by visual inspection as well as by radiographic and laboratory exams. At the start of the study, the two groups had no statistical differences concerning their clinical features: average age, gender, insulin use, diabetes mellitus onset time and previous amputations.

**Results**: PDT was effective in the treatment of 100% of the patients showing no relapses after one year of follow up. The group submitted to PDT without previous debridement had a statistically significant (p = 0.036, Mann-Whitney) shorter cure time (29 days, ~27%).

**Conclusion**: Our data indicates that debridement is not necessary in the treatment of diabetic foot in patients that have enough peripheral arterial perfusion. In addition, we reproduced previous studies confirming that PDT is an efficient, safe, simple and affordable treatment method for the diabetic foot.

## Introduction

Osteomyelitis usually responds poorly to antimicrobial therapy and infections starting in the toes can compromise the entire foot structure. Gangrene and osteomyelitis are two clinical situations with an indication for amputation. The susceptibility of diabetic patients to develop foot ulcers and osteomyelitis leads to a high risk of lower limb amputations, which is around 15–70 times higher than in non-diabetic patients [1–4].

The current consensus is that immediate debridement surgery is necessary as soon as the infection is clinically diagnosed [5]. Series of debridement procedures, which may be required to achieve complete cleaning of the wound, can take the medical doctor to decide for amputation [4]. Therefore, debridement surgery and amputation are justified in order to save the patient. A typical example is the transmetatarsal amputation, which leads to the maintenance of plantar support allowing patients to walk without crutches or artificial leg [1–6]. Although being a recommended procedure, recent data and literature reviews question this procedure with tendency to disfavor its use in the treatment of the diabetic foot. [7–9]

Photodynamic therapy (PDT) is a promising therapy for the management of various types of tumors and infecting diseases. The action mechanism of PDT is based on the photo-activation of specific compounds, called photosensitizers, which trigger cell death and modulate the immune response [10,11]. As an anti-microbial therapy, PDT stands as a procedure that does not induce microbial resistance [11]. Tardivo and collaborators have shown that PDT can be very useful in the treatment of the infected diabetic foot, avoiding amputation surgeries and the indiscriminate use of antibiotics. Moreover, PDT is an ambulatory procedure, involving a lot less costs compared with treatment modalities that require hospitalization [12–14]. It is important to mention that other non-systemic treatment modalities with or without debridement have also been shown to be effective in the treatment of the diabetic foot [15,16].

The purpose of this study is to evaluate the need for debridement in patients with infected diabetic foot treated with PDT. We compared clinical results in two groups of patients: the group that underwent ulcer debridement before PDT (debrided patients), with the group that did not go through debridement (non-debrided patients).

## Materials and methods

### Casuistry

The present study was performed as a controlled clinical trial with 57 patients with infected diabetic foot. All patients were treated in the Diabetic Foot Ambulatory at Padre Anchieta Hospital, Faculdade de Medicina ABC, from March 2011 to April 2015. Patients were referred to the Diabetic Foot Ambulatory by vascular surgeons. We included patients with serious infected diabetic foot, grade III in the Wagner classification and with high amputation risk. Most patients had confirmed osteomyelitis diagnosis, as evaluated by clinical examination ‘probe to bone’, X-rays and laboratory tests. Patients with clinical signs of peripheral vascular insufficiency were excluded from this study; because, in these cases, revascularization must precede the PDT treatment [17]. White blood cell count, ESR (erythrocyte sedimentation rate), and CRP (C-reactive protein) were also part of the initial evaluation procedure.

Patients were divided in two groups. The group of non-debrided patients (NDP) consisted of 40 patients referred directly to receive PDT treatment without prior hospitalization and/or debridement. The control group was named debrided patients (DP) and consisted of 17 patients that underwent debridement treatment previous to PDT. Table 1 shows the main clinical characteristics of the patients included in the present study. The average age of all 57 patients was 59.5 years (±9.6). The time of diabetes mellitus (DM) onset was 16.9 and 11.8 years in the DP and NDP groups, respectively; 70% and 57% of the patients in the DP and NPD groups, respectively, had insulin dependence. Among all 57 patients, 41.3% (58.8% in DP and 37.5% in NDP groups) had already had one or more minor amputations, such as fingers or transmetatarsal amputations. All patients were informed about the PDT treatment and signed the informed consent form. The FMABC Ethics Committee, case number 257/2010, approved this study.

**Table 1.** Features of the two study groups: non-debrided patients (NDP) and debrided patients (DP).

	NDP	DP	Total cases
Average age (years)	58	61	59
Median age (years)	57	61	59
Male gender (%)	65	70.6	65.2
Female gender (%)	35	29.4	34.8
Insulin use (%)	57.5	70.6	65.2
Non-insulin use (%)	42.5	29.4	34.5
Average of diabetes mellitus onset (age)	11.8	16.9	13.2
Previous amputations (%)	37.5	58.8	41.3

### PDT intervention protocol

**Figure 1.**
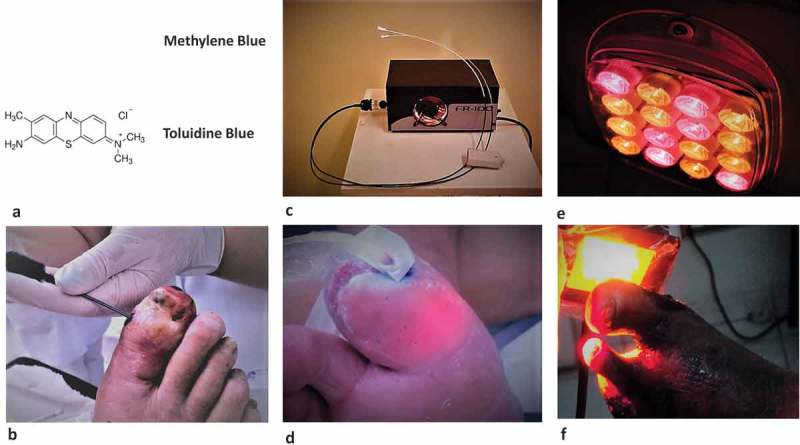
Steps in the treatment of the diabetic foot by PDT. (a) Molecular structure of the photosensitizers methylene blue and toluidine blue. A (1:1) mixture of two aqueous solutions containing each 1% (mass:mass) of the dyes was used in the treatments. (b) The photosensitizer solutions were applied topically and also injected in the ulcers. (c) A broadband emitter (400–725 nm, maximum at 560 nm) containing a white halogen light, was connected to optical fibers (1.0 or 1.5 mm), which were covered with clear PVC films and introduced in the ulcer cavities to allow efficient illumination of the infected sites. (d) Irradiation was performed weekly with a dose of 6 J/cm^2^. (e) Subsequently, a LED array with 590 nm and 640 nm centered LEDs was used to illuminate the surface of the infected sites. (f) Irradiations were performed either in close proximity to the lesion or 3–5 cm away from the feet always with a dose of 30 J/cm^2^. For further details please refer to the Methods section.

PDT sessions were held once a week. Stock solutions of the photosensitizers methylene blue and toluidine blue (Labsynth, São Paulo, Brazil) were prepared at the concentration of 1% (mass:mass). These solutions presented maximum value of light absorption at 664 nm and 630 nm, respectively. All patients with osteomyelitis received a (1:1) mixture of the photosensitizer solutions, via fistulas or ulcers, using syringes and catheters to enhance infiltration of the photosensitizer in the infected area, to stain the tissues and the infected bones. Dressings with dry gauze and crepe bands were used to cover the lesions immediately after PDT. In the intervals between the PDT sessions, patients had their dressing changed daily. Only sunflower oil was applied to the wounds. All these procedures were performed in outpatient facilities. Figure 1 illustrates the equipment and methods used in the PDT treatment of the diabetic foot.

All patients were photographed at every session, using a Sony Cyber-shot DSC-W310 digital camera (Sony Corporation), to observe and to measure the evolution of the treated areas. X-rays were performed to evaluate bone lesions in the feet, at the beginning and at the end of the treatment. A patient was discharged from the treatment after several clinical signs of cure, including ulcer healing, absence of clinical signs of inflammation and infection, improvement in laboratory tests and radiological improvement of the bone lesion. Patients were followed for 1 year after the PDT treatment, in order to evaluate any new event on their feet.

All patients received daily systemic oral antibiotics, 1 g of ciprofloxacin and 900 mg of clindamycin during the first 14 days of treatment. The main objective of the use of systemic antibiotics was to avoid the spread of the infection. PDT has local and non-systemic action. Therefore, once the purulent secretions stopped being produced in the wound, the systemic antibiotics were suspended in order to preserve renal function and to prevent the development of bacterial resistance.

Two light sources were used. The first was a FR-100 white halogen light source model (FASA Ind. Com. Imp., São Paulo, Brazil), with optical fibers 1.0 and 1.5 mm in diameter and 1 m long. This light source is a broadband emitter, whose spectrum covers completely the visible range (400–725 nm) with maximum at 560 nm and with at least 30% of the spectral power in the biological window (visible red with wavelengths above 600 nm). FR-100 was calibrated using a spectrophotometer surface model USB2000 (Ocean Optics, Florida, USA). Optical fibers were introduced through external fistulas immediately after staining the cavitary lesions by infusion of the phenothiazine solutions. The ends of the fibers were suitably covered with a clear PVC film, previously washed with aqueous chlorhexidine (Riohex, Farmacêutica Rioquímica LTDA, São José do Rio Preto, SP). Irradiation dose was of 6 J/cm^2^ per fiber, for 10 minutes. This procedure was repeated weekly. The second light source consisted of a light-emitting diode (LED) array (GDE Genesis LEDs Solutions Ind., São Paulo, Brazil) comprising LEDs with maximum emission spectrum at 590 nm and at 640 nm. Irradiation was performed at 50 mW output power and dose of 30 J/cm^2^ for 10 minutes. The LED source was always placed in contact with the surface of the foot separated by a thin sheet of transparent and sterile plastic or placed at a distance of 3–5 cm from the irradiated surface.

### Statistical analysis

Qualitative variables were described by absolute and relative frequencies and quantitative values were expressed as the mean and standard deviation or as the median and range. Frequency comparisons among groups were performed with chi-square test or Fisher’s exact test. The distributions of quantitative variables were defined using a nonparametric Kolmogorov–Smirnov test and a Mann–Whitney test was used to compare independent groups. To verify the correlation between two quantitative variables, it was used the Spearman correlation test. In all analyzes, SPSS statistical software, version 17.0 (SPSS Inc, Illinois, USA) was used, and statistical significance level of *p* < 0.05 was adopted in all comparisons.

## Results

From the 57 patients included in this study 40 received PDT treatment without any debridement (NDP) and 17 were initially debrided to receive PDT subsequently (DP). In order to check if the DP and NDP study groups were comparable, we analyzed the use of insulin, gender, previous amputations, average age and the diagnostic time of DM, as shown in Tables 1 and 2. Although, the average time of diabetes in the DP group was a bit longer than in the NDP group (16.9 years compared 11.5 years), there was no statistical difference between these groups regarding the diabetes time. In fact, there was no significant difference between the two groups in any of the variables used to define the patient conditions before the start of PDT (Table 2). We must emphasize that the photodynamic effect depends on oxygen and patients having peripheral arterial disease (PAD) must be initially submitted to a revascularization procedure before PDT [17]. Therefore, patients with severe PAD were excluded from this study.

**Table 2.** Statistical comparison between DP and NDP groups before PDT treatment.

Variables	Total (*n* = 57)	DP (n = 17)	NDP (n = 40)	*p* (test)
Insulin use	35/56 (62.5%)	12/17 (70.6%)	23/39 (59.0%)	0.551 (Fisher’s exact)
Male gender	38/57 (66.7%)	12/17 (70.6%)	26/40 (65.0%)	0.766 (Fisher’s exact)
Amputations	24/57 (42.1%)	9/17 (52.9%)	15/40 (37.5%)	0.280 (chi square)
Age (years)	59.5 ± 9.6	61.1 ± 9.6	58.6 ± 9.6	0.427 (Mann–Whitney)
DM (ages)	13.2 ± 8.6	16.9 ± 10.0	11.6 ± 7.5	0.066 (Mann–Whitney)

DM, diabetes mellitus.

**Figure 2.**
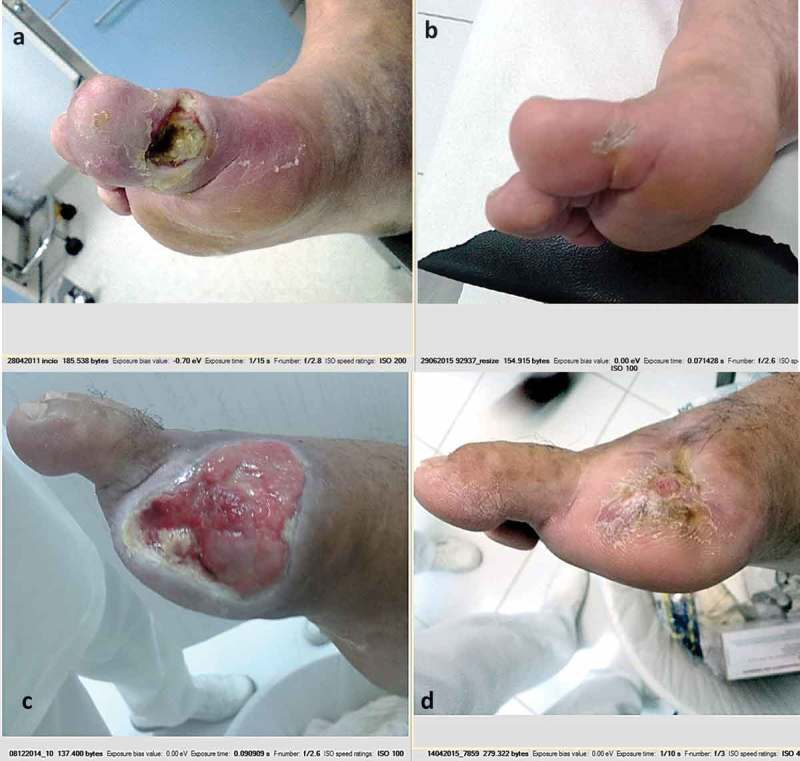
Images of patients before and after PDT. (a, b) Patient from the NDP group, was treated for 62 days to complete healing (28/04–29/06). (c, d) Patient from the DP group, had his lesion healed in 125 days (08/12–14/04).

**Figure 3.**
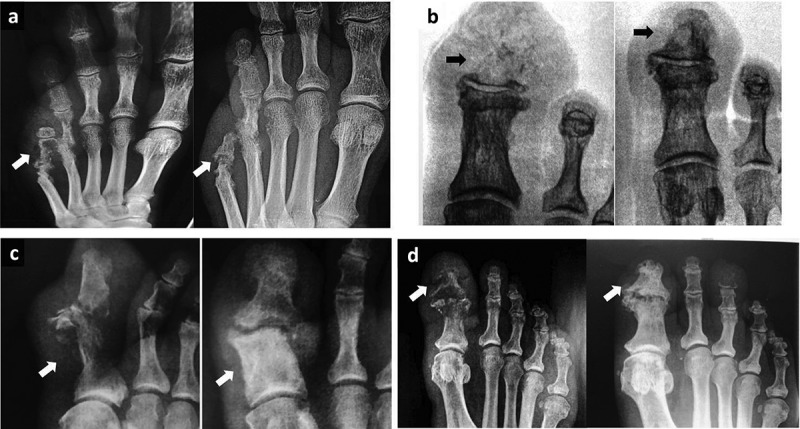
Osteomyelitis before and after PDT. X-Ray images obtained from patients with osteomyelitis before and after PDT treatment. A, B, C, and D are X-ray images from four patients treated with PDT. Arrows at left are showing osteomyelitis in metatarsal and phalanges before treatment with cortical disruption and pathological destruction or disappearance of bone tissue. Arrows at right represent the same patients after PDT. Note the effective regeneration of diseased bones after PDT. A and B were obtained from patients of the DP group, C and D from the NDP group.

All patients were treated with PDT and had their ulcers healed, i.e., remission of osteomyelitis was observed in 100% of the cases in both DP and NDP groups. Figure 2 shows two typical cases. Complete bone reconstruction was observed through radiological images, as shown in Figure 2. In many cases, there was formation of bone callus in regions that had previously suffered fractures or lysis of bones (Figure 3). No patient had adverse reactions from PDT [12,13,17].

Table 3 shows that the healing time after PDT varied significantly between the two groups. Patients in the DP group took an average of 135 ± 67 days for healing, while in the NDP group 106 ± 77 days. The difference in healing time was statistically significant (*p* = 0.036). An amputation occurred in the group of patients who underwent debridement before treatment with PDT, but it represented just one case (Table 3). Clearly, PDT performed better with faster healing time (29 days shorter) in patients of the NDP group. Statistical analyzes (Spearman’s correlation test) show no correlation between the time required for treatment versus the previous diabetic period (*r* = 0.177, *p* = 0.206); nor between treatment time versus number of amputations (*r* = 0.103, *p* = 0.444), nor there is correlation between the time of diabetes versus the number of amputations (*r* = 0.221; *p* = 0.111). Therefore, we can only attribute the 27% increase in the healing time to the initial debridement procedure.

**Table 3.** Statistical comparison between DP and NDP groups after PDT treatment.

Variables	Total (*n* = 57)	DP (*n* = 17)	NDP (n = 40)	*p* (test)
Time of PDT (days)	114.5 ± 74.5	135.1 ± 66.7	105.8 ± 76.7	0.036 (Mann–Whitney)
Number of amputations (median; amplitude)	0 (0–3)	1 (0–3)	0 (0–3)	0.429 (Mann–Whitney)

PDT, photodynamic therapy.

## Discussion and conclusions

Patients that live in regions with poor health care system have more risks of complications from the classic debridement procedure, such as infections and general worsening of prognosis. The group of patients who previously suffered debridement required an average of 29 extra days to cure (Table 3), compared with the group that went directly to PDT. Therefore, sparing debridement procedure in diabetic patients undergoing PDT will shorten the healing period. The quicker treatment of the diabetic foot results in better outcome for the patient, lower involved costs, and possibly lower risks of amputation.

Although these results were obtained with a highly efficient antimicrobial intervention (PDT), it is likely that the debridement procedure may not be recommended for any patient with reasonable peripheral blood perfusion that is being treated with any local antimicrobial intervention.^12,17^ This is in agreement with recent literature data that disfavor debridement in the treatment of the diabetic foot in comparison with other procedures [8,9]. Our work was based in a controlled study group, with photographic and radiographic follow up for over one year. We hope this data can provide support to justify a larger non-randomized double blind clinical trial.

Diabetic patients with osteomyelitis, without signs of ischemia have excellent chances of cure with PDT, without the need of amputation or of extensive debridement procedures. PDT confirmed to be a safe and low-cost outpatient treatment, showing effective results for the treatment of the diabetic foot.
